# Load-Dependent Increases in Delay-Period Alpha-Band Power Track the Gating of Task-Irrelevant Inputs to Working Memory

**DOI:** 10.3389/fnhum.2017.00250

**Published:** 2017-05-15

**Authors:** Andrew J. Heinz, Jeffrey S. Johnson

**Affiliations:** ^1^Department of Psychology, North Dakota State UniversityFargo, ND, USA; ^2^Center for Visual and Cognitive Neuroscience, North Dakota State UniversityFargo, ND, USA

**Keywords:** working memory, neural oscillations, alpha-band activity, EEG, distractor processing

## Abstract

Studies exploring the role of neural oscillations in cognition have revealed sustained increases in alpha-band power (ABP) during the delay period of verbal and visual working memory (VWM) tasks. There have been various proposals regarding the functional significance of such increases, including the inhibition of task-irrelevant cortical areas as well as the active retention of information in VWM. The present study examines the role of delay-period ABP in mediating the effects of interference arising from on-going visual processing during a concurrent VWM task. Specifically, we reasoned that, if set-size dependent increases in ABP represent the gating out of on-going task-irrelevant visual inputs, they should be predictive with respect to some modulation in visual evoked potentials resulting from a task-irrelevant delay period probe stimulus. In order to investigate this possibility, we recorded the electroencephalogram while subjects performed a change detection task requiring the retention of two or four novel shapes. On a portion of trials, a novel, task-irrelevant bilateral checkerboard probe was presented mid-way through the delay. Analyses focused on examining correlations between set-size dependent increases in ABP and changes in the magnitude of the P1, N1 and P3a components of the probe-evoked response and how such increases might be related to behavior. Results revealed that increased delay-period ABP was associated with changes in the amplitude of the N1 and P3a event-related potential (ERP) components, and with load-dependent changes in capacity when the probe was presented during the delay. We conclude that load-dependent increases in ABP likely play a role in supporting short-term retention by gating task-irrelevant sensory inputs and suppressing potential sources of disruptive interference.

## Introduction

Our ability to selectively attend to and remember goal-relevant information while ignoring potentially distracting information is essential to the stability and efficacy of goal-oriented cognitive functioning. Such cognitive control processes are thought to be mediated by neural mechanisms underlying cortical oscillations across a variety of frequency bands (Klimesch et al., 1997; Başar et al., 2001; Buzsáki, 2006) In particular, electroencephalographic (EEG) recording studies in humans have suggested that elevated power in the alpha frequency band (~8–12 Hz) potentially reflects the operation of an inhibitory mechanism that serves to suppress the neural representation of task-irrelevant and potentially distracting information (Fu et al., 2001; Jensen et al., 2002; Klimesch et al., 2007; Jensen and Mazaheri, 2010; Dube et al., 2013; Payne et al., 2013) while simultaneously serving to help route relevant inputs through task-relevant networks. Supporting this possibility, the scalp distribution of alpha-band power (ABP) has been observed to covary with the focus of attention, increasing over brain areas representing task-irrelevant information (e.g., spatial locations, sensory modalities, feature dimensions, etc.), and decreasing over task-relevant areas (Worden et al., 2000; Fu et al., 2001; Snyder and Foxe, 2010; Jensen et al., 2012; Klimesch, 2012; Dube et al., 2013; Payne et al., 2013).

Similar modulations of ABP have also been observed during the delay period of working memory tasks (Jokisch and Jensen, 2007; Van Der Werf et al., 2008; Grimault et al., 2009; Sauseng et al., 2009; Poch et al., 2014), prompting the proposal that alpha oscillations may reflect the operation of an inhibitory mechanism in this case as well. However, these increases in delay-period ABP have also been observed within brain areas representing task-relevant information (Lopes da Silva, 1991; Cooper et al., 2003; Grimault et al., 2009; Jensen and Mazaheri, 2010; Johnson et al., 2011; Mo et al., 2011; Bonnefond and Jensen, 2012), in some cases scaling as a function of the amount of relevant information held in working memory (Jensen et al., 2002; Osipova et al., 2006; Medendorp et al., 2007; Tuladhar et al., 2007; Mo et al., 2011; Palva et al., 2011; Honkanen et al., 2015; Poch et al., 2014). These findings have raised questions about the generalizability of any purely inhibitory interpretation, prompting the alternative proposal that elevated delay-period ABP may reflect a critical component of the distributed network activity underlying the selection and maintenance of objects in working memory, rather than inhibition (Jensen et al., 2002; Cooper et al., 2003; Grimault et al., 2009; Bonnefond and Jensen, 2012).

Alternatively, however, these observations could be reconciled with a version of previously proposed inhibitory accounts (Klimesch et al., 2007; Jensen and Mazaheri, 2010; Klimesch, 2012; Samaha et al., 2015) if, in some cases, rather than being directed exclusively toward specific task-irrelevant properties of attended stimuli, increased delay-period ABP reflected a more general mechanism of gating aimed at inhibiting an array of processes not relevant to the current task. As in these other cases, this would, by extension, bias activation in favor of relevant processes. For example, the load-dependent increases in delay-period ABP observed over more posterior visual areas could reflect the active suppression of task-irrelevant on-going visual inputs, i.e., those not matching the contents of visual working memory (VWM). This would serve to support maintenance by insulating target representations against disruption via bottom-up interference. By this view increases need not represent the items *per se* in order to support VWM maintenance.

To investigate these alternative proposals, we recorded EEG during the performance of a VWM task requiring the retention of either two or four abstract shapes. Critically, on a subset of trials, a task-irrelevant probe stimulus was presented midway through the memory retention interval to gauge cortical excitability with respect to on-going visual sensory processing. We hypothesized that if load-dependent increases in delay-period ABP reflect an inhibitory mechanism serving to gate irrelevant inputs at one or more levels of visual processing, they should be associated with a reduction in specific components of the visual evoked potential elicited by the task-irrelevant probe stimulus. More specifically, if delay-period ABP reflects the inhibition of initial sensory processing of the probe, we expected to observe a correlation between set-size dependent increases in delay-period ABP and set size-dependent suppression of the amplitudes of early event-related potential (ERP) components, such as the P1 and N1. Alternatively, increases might reflect the gating of visual processing at some later point along the visual processing hierarchy. If this were the case, we expected set-size dependent changes in delay-period ABP to be correlated with the modulation of later ERP components related to the capture of attention, such as the anterior P3a.

Moreover, within-participant variability in the strength of delay-period ABP should be predictive of the disruptive effects of the probe stimulus on performance in the change detection task. By contrast, if delay-period ABP reflects neural processes underlying the short-term retention of information in working memory *per se*, rather than inhibition, we expected delay-period ABP to scale with the amount of information held in working memory even when no disruptive probe stimulus is presented.

Finally, to investigate the possible role of top-down feedback from frontal control areas in mediating changes in delay-period ABP, we calculated inter-site phase clustering (ISPC), a measure of functional connectivity between cortical areas (Cohen, 2014). In particular, we were interested in determining whether ISPC between frontal and posterior electrode sites increases as a function of WM load and whether such changes are predictive of load-dependent changes in delay-period ABP. Prior evidence suggests that control processes engaged during attention and working memory tasks such as these likely rely on functional interactions between frontal and more posterior/parietal regions (Miller et al., 1996; Prabhakaran et al., 2000; Rowe et al., 2000; Sakai et al., 2002; Warden and Miller, 2010; Rigotti et al., 2013; Sreenivasan et al., 2014). Of particular relevance, it has been suggested that increased inter-areal phase synchronization in the alpha band may reflect the coordination of activity within distributed cortical cell assemblies relevant to maintaining items in VWM (Jensen et al., 2002; Sauseng et al., 2009; Palva et al., 2010). If this were the case, we expected ISPC to increase with load and to predict the load-dependent changes in delay-period ABP.

## Materials and Methods

### Participants

Twenty-five participants between the ages of 18 and 35 were recruited from the North Dakota State University student population. All participants reported normal or corrected to normal visual acuity, and provided written informed consent prior to participation in accordance with the Declaration of Helsinki. Participants received either course credit or monetary compensation ($15/h) for their participation. Two participants were not included in the analysis for failing to complete all of the experimental blocks and five additional were dropped due to excessive EEG artifacts, resulting in a total of 18 participants. All experimental protocols were approved by the North Dakota State University Institutional Review Board (IRB).

### Stimuli and Procedures

During the experiment, participants were seated in a dimly lit, noise-controlled room. Stimulus presentation and response collection was controlled by a PC running Presentation software (Neurobehavioral Systems, Inc.). Stimuli were presented against a light gray background (RGB = [125, 125, 125]) on the surface of a 19″ cathode ray tube monitor with a refresh rate of 100 Hz, at a viewing distance of 70 cm.

**Figure 1:**
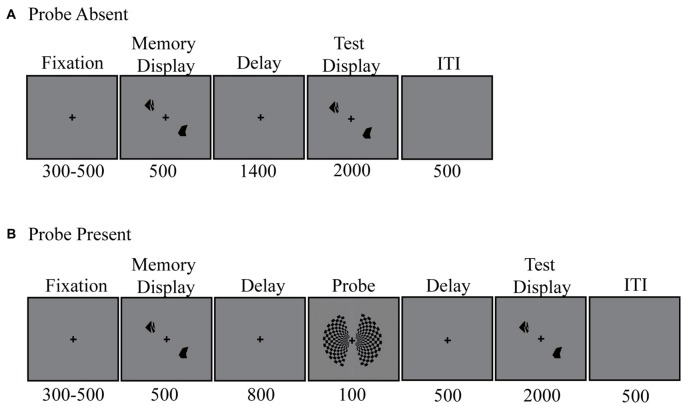
**Delayed recall task. (A)** Probe absent condition. **(B)** Probe present condition.

Participants were asked to perform a change detection task (Figure 1). Specifically, they were instructed to maintain either two or four target shapes in memory across a 1400-ms unfilled delay. Each trial began with the appearance of a centrally presented fixation cross. Participants were asked to maintain focus on this fixation cross for the duration of each trial. Three-hundred to five-hundred milliseconds after fixation onset, the memory display appeared. Target items were presented bilaterally around the fixation point, in one of eight possible locations; four per side. The memory display remained visible for 500 ms. Memory target items consisted of black abstract shapes (Attneave and Arnoult, 1956), drawn at random from among 12 such shapes.

Randomly, on two-thirds of trials, a bilateral checkerboard probe stimulus was presented 800 ms into the delay period, for 100 ms. Participants were instructed to ignore this stimulus. The squares within the checkerboard alternated between black and light gray (RGB = [125, 125, 125]).

Following the delay period, participants reported whether or not any of the items within the test display had changed shape relative to those in the memory display. When a change occurred, one of the shapes was replaced by a different shape not present in the original memory display. To constrain the magnitude of shape changes across trials, the 12 shapes were grouped into four subsets of three items, based on experimenter estimates of subjective similarity (e.g., more elongated vs. more rectangular). Shapes could change from a member of one subset to another, but never to a new shape within the same subset. Participants responded by making a “change” or “no-change” response using one of the response buttons held in their left and right hands. The test display remained visible for 2 s or until a response was made. Feedback was given on every trial and remained visible for 500 ms. For correct responses, the fixation cross briefly changed to a bold font. In contrast, when either an incorrect response was given or 2 s had passed with no response, the fixation cross changed to a minus sign. Participants completed a total of 36 trials per block and 20 blocks in all. This amounts to 480 probe trials (240 per set size tested) and 240 no-probe trials (120 per set size).

To estimate participants’ WM capacity, a standard formula described by Cowan (2001) was used. Specifically, for each set-size (2, 4) and probe (present, absent) condition, WM capacity (K) was calculated using the formula K = set size × (hit rate−false alarm rate). K is a common metric utilized within the VWM literature that allows for comparisons across various set sizes.

### EEG Acquisition and Preprocessing

EEG was recorded using active Ag/AgCl electrodes (BioSemi Active Two) positioned at the left and right mastoids and 64 scalp sites, according to the modified international 10–20 system (American Electroencephalographic Society, 1994). To detect eye movements and blinks, the electrooculogram (EOG) was recorded from electrodes placed at the outer canthi of each eye, as well as above and below each eye. All signals were recorded with a band-pass of 0.01–100 Hz and a sampling rate of 512 Hz.

Data was processed offline using the EEGLab (Delorme and Makeig, 2004) and ERPLab (Lopez-Calderon and Luck, 2014) open source Matlab-based toolboxes, and custom analysis scripts are written in Matlab (MathWorks, Inc., Natic, MA, USA). The EEG and EOG signals were band-pass filtered using a non-causal Butterworth infinite impulse response filter with half-amplitude cut-offs of 0.1 and 30 Hz (12 dB/octave roll-off) and re-referenced to the average of the mastoid electrodes. The data were epoched into 2500-ms segments spanning the interval 500 ms before to 2000 ms after memory display onset (100 ms after test display onset).

The EOG signals were referenced into bipolar vertical and horizontal derivations and used in the detection of eye blinks and saccades. Trials were automatically excluded if EEG amplitude exceeded 100 μV in any channel (or 70 μV in the vertical EOG channel) within a moving window of 200 ms. Trials were also rejected if a step function detected changes of more than 25 μV on the horizontal bipolar EOG channel, indicating the presence of lateral eye movements. Participants were excluded from subsequent analyses if the total number of rejected trials exceeded 30% of total trials. Five participants were excluded for this reason.

### EEG Analyses

#### Time-Frequency Analysis of Power

Time-frequency decomposition of EEG signals was performed using the Fieldtrip software package (Oostenveld et al., 2010), an open-source Matlab-based toolbox for the analysis of electrophysiological data, and custom scripts written in Matlab. Time-frequency representations (TFRs) were obtained by convolving stimulus-locked single-trial data from all electrodes with complex Morlet wavelets: $e^{i2\pi ft}e^{-t^{2}}/(2\sigma^{2})$ where *t* is time, *f* is frequency, which varied from 4–40 Hz in 37 logarithmically spaced steps, and σ is the width of each frequency band, defined as *n*/(2π*f*), where *n* is the number of wavelet cycles, which varied from 3–6 in logarithmically spaced steps to obtain comparable frequency precision at low and high frequencies. TFRs of power were estimated by squaring the complex convolution signal *Z* (*power* = real[*z*(*t*)]^2^ + imag[*z*(*t*)]^2^) and averaging across trials. Power values at each time-frequency point were normalized by converting to the decibel scale using the formula 10log_10_(*power/baseline*), with the −300 to −100 ms pre-stimulus interval used as the frequency band-specific baseline. This is a relative baseline measure that accounts for power-law scaling of power in different frequencies.

#### Probe-Evoked Response Analyses

To quantify the probe-evoked response (p-ER), artifact-free trial epochs created during preprocessing were used to compute averaged ERP waveforms within a narrower time window. Specifically, the size of each trial epoch was reduced to encompass a 600-ms time window, ranging from 100 ms before to 500 ms after probe onset. Individual trials were baseline corrected using the 100 ms pre-probe interval. Time-domain analysis focused on comparing the mean amplitude of specific components of the p-ER, including the posterior P1 (75–125 ms) and N1 (125–175 ms) components, as well as the later anterior visual P3a (250–400 ms) component observed over fronto-central electrode sites. Specifically, for each load condition (2 or 4) mean amplitude was calculated in the time range of the P1 and N1 at two electrode sites (PO7/PO8) where these components were maximal across conditions. Similarly, the local (positive) peak amplitude at frontal midline electrode FCz, during the P3a latency range was averaged across trials. This alternative method of estimating P3a amplitude was used in an effort to disentangle the distinct positive and negative components underlying the overlapping N2 and P3a waveforms as well as to avoid obscuring potential modulations by averaging across the wide range of possible latencies ascribed to the P3a. This choice of analysis parameters is consistent with both the range of the latencies and topographic distribution commonly ascribed to these components (Polich, 2007; Folstein and Van Petten, 2008).

#### Phase-Based Connectivity Analyses

To examine load-specific differences in oscillatory phase coupling between frontal and posterior brain areas, a measure of phase coherence known as ISPC was used. ISPC reflects the degree to which the phase angle differences between a given pair of electrodes are clustered in polar space, at a specific time point, across trials. This specific measure was chosen for two reasons. First, it provides relatively strong evidence for task-related modulations in connectivity of the kind we expected to observe across load conditions. Second, this method allows for a relatively high degree of temporal precision as compared to other similar methods. The analysis was conducted using custom scripts written in Matlab, following the procedure described in Cohen (2014).

Prior to ISPC analysis, a surface Laplacian was applied to EEG data epochs (Perrin et al., 1989). The surface Laplacian is a spatial bandpass filter that attenuates low spatial frequencies, which helps to minimize spurious connectivity effects arising from volume conductance, and renders the data more appropriate for electrode-level connectivity analysis (Cohen, 2014).

Following application of the surface Laplacian, single-trial EEG epochs were decomposed into their constituent TFRs by convolving them with a set of Morlet wavelets, as described above, with frequencies ranging from 8 Hz to 30 Hz, in 14 logarithmically spaced steps, and number of wavelet cycles varying from 4 to 8 in logarithmically spaced steps. Frequency-band specific ISPC was computed using the phase angle, *φ_t_* = arctan (imag[*z*(*t*)]/real[*z*(*t*)]), of the complex convolution result. ISPC is defined as the trial-averaged phase angle difference between two electrodes *j* and *k* at each time-frequency point:

$$\left|\frac{1}{n}\sum_{t=1}^{n}e^{i(\varphi jt-\varphi kt)}\right|$$

where *n* is the trial number, *j* is the seed electrode and *k* is the set of all other electrodes. The frequency-specific average of ISPC values over the –300 to –100 ms interval prior to the memory display was used for baseline correction. Prior evidence suggests that cognitive-control related modulations of ERPs, oscillatory power and connectivity have been observed over the mid-frontal cortex (Gulbinaite et al., 2014). We therefore chose to focus on FCz, which is centered over these regions, as a seed electrode for this analysis. Although we calculated ISPC between FCz and all other electrodes, our correlation analysis focused specifically on POz and Pz, as this is where the load-dependent differences in delay-period ABP were observed. We therefore reasoned that if ISPC reflects a control processes driving these increases we would expect to observe relatively high phase clustering between these two sets of electrodes.

## Statistical Analyses

### Behavioral Data

To examine load- and probe-related changes in capacity (K) we conducted a two-way repeated-measures ANOVA with factors of set size (2, 4) and probe (present, absent). Additionally, to examine the relationship between observed changes in capacity across conditions and changes in spectral properties of the EEG (e.g., delay-period ABP), correlation analysis was conducted using Pearson’s *r* (described further below).

### EEG Data

A critical assumption of the present study is that delay-period ABP will increase as a function of WM load (set-size). Thus, the first step in the EEG analysis was to determine whether this was, in fact, the case. To do this, estimates of delay-period ABP, obtained using the wavelet method described above, were averaged across time for the portion of the delay during which above baseline delay-period alpha band power was generally observed independently across all conditions (1000–1800 ms). Next, the resulting values for the interval encompassing the 100 ms prior to the probe were compared across conditions and set-sizes using a two-way repeated-measures ANOVA with factors of set-size and probe condition. Importantly, since there were twice as many trials in the probe-present conditions, we randomly selected only a subset of these trials to match trial numbers across conditions before carrying out this comparison. Lastly, load-dependent changes in the p-ER were assessed via three separate paired sample *t*-tests comparing the mean amplitude of the P1, N1 and P3a across load conditions.

To examine the relationship between delay-period ABP and the p-ER, correlation analysis (Pearson’s *r*) was used to determine whether observed load-dependent differences in delay-period ABP in the interval prior to probe presentation were predictive of load-dependent changes in the p-ER. If delay-period ABP reflects the inhibition of initial sensory processing of the probe, we expected to observe a correlation between set-size dependent increases in delay-period ABP and set size-dependent suppression of the amplitudes of early ERP components, such as the P1 and N1. Alternatively, if increased ABP reflects the gating of visual processing at some later point in the visual processing hierarchy, we would expect observed load-dependent increases to be correlated with the modulation of later ERP components, such as the anterior N2 or P3a components. Whereas the anterior N2 has been associated with the degree of mismatch between sequentially presented stimuli, the P3a has been shown to be more sensitive to involuntary attentional orienting to novel stimuli in the environment, and, more generally, to the degree of distraction from a mental set (see reviews in Polich, 2007; Folstein and Van Petten, 2008). Given these differences, we chose to focus on the P3a.

To examine the relationship between load-dependent changes in delay-period ABP and any load- and probe-related changes in WM capacity, we first calculated the change in capacity as a function of set size (K_SS4_−K_SS2_) for each participant, and used these change values to sort participants into separate High and Low capacity change groups using a median split. We then performed an independent samples *t*-test to assess differences in observed load-dependent changes in delay-period ABP across these groups. Additionally, to investigate the possibility that load-dependent increases in delay-period ABP reflect inhibition rather than maintenance of target items, we conducted a second set of analyses in which we once again sorted participants into separate High and Low K groups using a median split; in this case, based on the observed load-dependent changes in K across set-sizes in the probe-present condition. An independent samples *t*-test was then performed, comparing the difference in pre-probe delay-period ABP across these groups.

Lastly, to assess whether observed load-dependent increases in delay-period ABP were a consequence of an attentionally selective frontal control process, Pearson’s *r* was calculated to assess the correlation between set-size dependent increases in ISPC and temporally coincident modulations of delay-period ABP. If set-size dependent increases in delay-period ABP are the consequence of an executive control mechanism, one would expect to observe a positive correlation between increases in delay-period ABP and ISPC prior to the probe.

## Results

### Behavior

**Figure 2:**
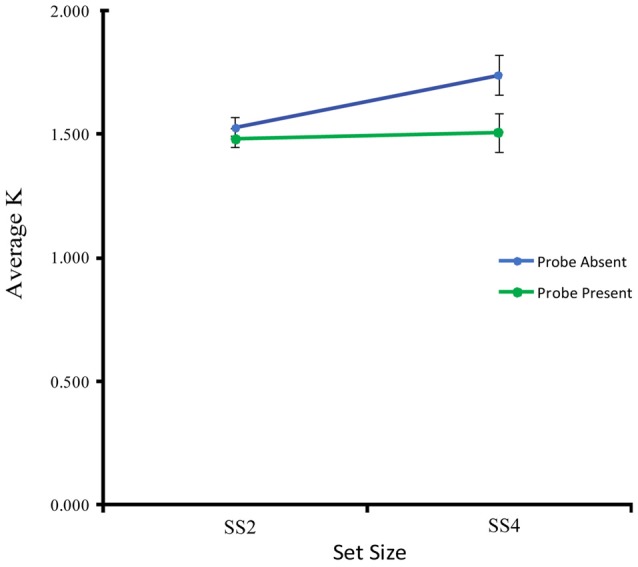
**Estimated visual working memory capacity (K) averaged across subjects for each set size and probe condition**.

The behavioral results, highlighted in Figure 2, show that on average participants were able to maintain the same number of items at SS2 in both the probe-present and probe-absent conditions. At SS4, however, participants exhibited an increase in VWM capacity in the probe-absent, but not the probe-present, condition, suggesting that maintenance may have been disrupted by the probe stimulus at higher loads. A two-way (2 probe conditions × 2 set sizes) within-participants analysis of variance exploring these effects yielded a main effect of probe condition, *F*_(1,17)_ = 11.29, *p* = 0.004, $\eta_{\text{p}}^{\text{2}}$ = 0.39, such that average K was significantly higher in the probe-absent (*M* = 1.61, *SD* = 0.37) vs. probe-present condition (*M* = 1.49, *SD* = 0.34). There was also a trend towards a main effect of set size, *F*_(1,17)_ = 4.21, *p* = 0.056, $\eta_{\text{p}}^{\text{2}}$ = 0.20, and a trend towards an interaction, *F*_(1,17)_ = 4.01, *p* = 0.062, $\eta_{\text{p}}^{\text{2}}$ = 0.19. Follow-up *t*-tests comparing each specific load- and probe-related change in capacity (K) across conditions, revealed that the probe significantly disrupted performance at SS4, *t*_(17)_ = −2.92, *p* = 0.004, *d* = 0.69, but not at SS2, *t*_(17)_ = −1.257, *p* = 0.112, *d* = 0.30. Further, participants were able to maintain significantly more information in the SS4 as compared with the SS2 condition when the probe was not presented, *t*_(17)_ = 2.42, *p* = 0.019, *d* = 0.57, but did not differ significantly with set size when the probe was presented, *t*_(17)_ = 0.302, *p* = 0.383, *d* = 0.07.

### EEG

#### Delay-Period ABP Differences

**Figure 3:**
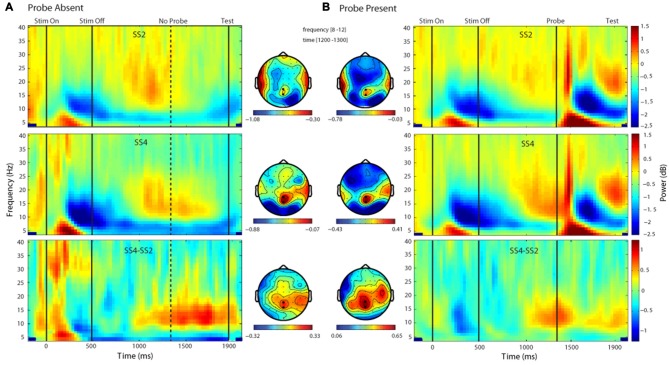
**Time-frequency and topographic plots of delay period alpha-band power (DPABP).** Each panel depicts oscillatory power across the delay interval and the associated topographic distribution of ABP averaged across the 1300–1400 ms interval, for set size 2, 4 and their difference (SS4-SS2) in the Probe Absent **(A)** and Probe Present **(B)** conditions. Emboldened electrode markers (black dots) reflect the electrodes over which power was averaged for the present analysis.

Time-frequency plots depicting changes in oscillatory power for each probe condition (absent, present) and set size (2, 4) are shown in Figure 3. As can be seen in Figure 3A, in the absence of a probe, there was an apparent load-dependent increase in oscillatory power in a band spanning the alpha and low beta frequencies and extending from ~1000 ms after memory display onset until the end of the delay. A *t*-test of the difference in the alpha band (8–12 Hz) averaged over the interval 1000–1800 ms post-memory display onset, confirmed that this difference was significant, *t*_(17)_ = 2.26, *p* = 0.037, *d* = 0.53. To further examine load-dependent increases in the probe-present condition, we conducted an additional *t*-test comparing delay-period ABP across loads, this time focusing on the 100-ms time window just prior to probe onset, rather than the bulk of the delay. A *t*-test confirmed that during the 100 ms prior to probe onset there was a significant increase in delay-period ABP across set sizes at electrodes POz and Pz, *t*_(17)_ = 3.38, *p* = 0.0036, *d* = 0.80.

Finally, we compared delay-period ABP across set-sizes and conditions. As can be seen in Figures 3A,B, the change in ABP across the delay period followed a similar time course and topography in each condition; in general, increasing across set-sizes. A two-way (2 probe conditions × 2 set sizes) within-participants ANOVA exploring these effects yielded a main effect of set size, *F*_(1,17)_ = 10.31, *p* = 0.005, $\eta_{\text{p}}^{\text{2}}$ = 0.377, such that average ABP was significantly higher in the SS4 vs. SS2 condition. There was also a main effect of probe, *F*_(1,17)_ = 6.76, *p* = 0.019, $\eta_{\text{p}}^{\text{2}}$ = 0.28, with overall greater power in the probe present vs. absent condition. The probe × set-size interaction did not reach significance *F*_(1,17)_ = 0.387, *p* = 0.542, $\eta_{\text{p}}^{\text{2}}$ = 0.022.

#### Probe-Evoked Response Differences

**Figure 4:**
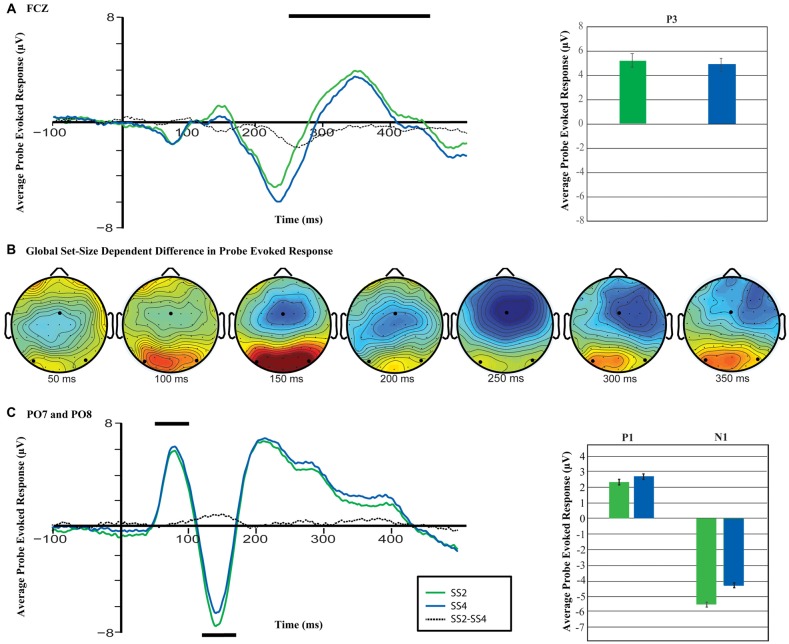
**Differences in the probe-evoked response (p-ER) across conditions. (A)** Anterior P3a wave for set size two and four averaged across trials for electrode FCz. The black line reflects the interval over which activity was averaged for the corresponding bar plots. **(B)** The difference in the p-ER across electrodes (SS4-SS2). Emboldened black dots mark the locations of electrodes FCZ, PO7 and PO8. **(C)** P1 and N1 waveform across set sizes. As before, the black line reflects the interval over which activity was averaged for the bar plots shown to the right, and the statistical analyses described in the text.

Figure 4 shows the topography and time-course of the probe-evoked response across load conditions. As can be seen, the probe elicited a robust early response in bilateral posterior sites centered on electrodes PO7 and PO8, and a later response centered over frontocentral electrode FCz. A comparison of the amplitude of the evoked response across set sizes at these separate pairs of electrodes revealed a significant load-dependent increase in the P1, *t*_(17)_ = 3.24, *p* = 0.004, *d* = 0.76, and a load-dependent decrease in the N1, *t*_(17)_ = 6.43, *p* < 0.001, *d* = 1.52 at PO7/8 (Figure 4C). Similarly, at electrode FCz, the analysis revealed a significant set-size dependent reduction in the amplitude of the p-ER in the time range of the P3a component (250–400 ms), *t*_(17)_ = 2.54, *p* = 0.021, *d* = 0.60. Thus, increased working memory load was related to reliable changes in the brain’s response to the visual-probe stimulus at multiple time points. It is worth noting that upon *post hoc* inspection there appear to be load-dependent differences in the ERP prior to as well as just following stimulus onset, which are of similar magnitude to the observed P1 modulations (see Figure 4C). This could explain the observed differences in the P1, but is unlikely to explain the observed differences in the N1, which is considerably larger than the pre-stimulus difference.

#### Relationship between Delay-Period ABP and p-ER

**Figure 5:**
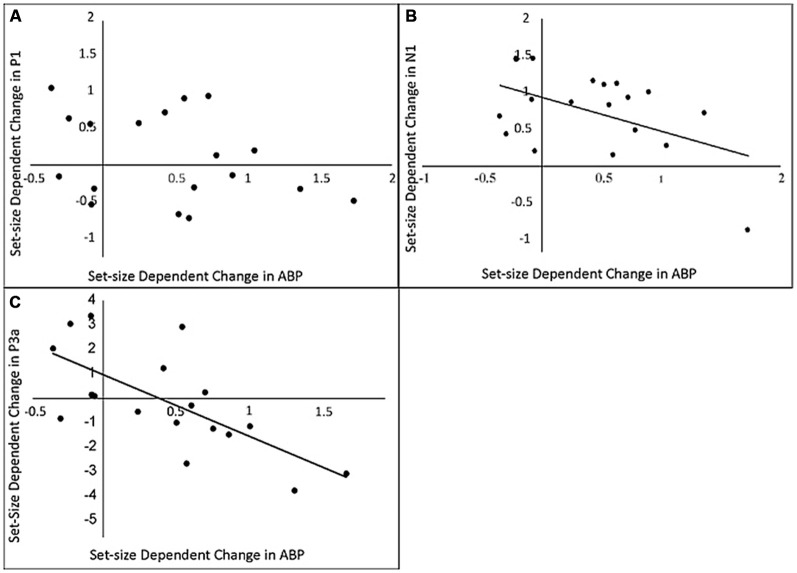
**The relationship between load-dependent change in delay-period ABP and load-dependent change in amplitude of P1 (A)** N1 **(B)** and P3 **(C)** components of the probe-evoked response.

The next step in our analysis was to determine whether the observed load-dependent changes in the p-ER were systematically related to differences in delay-period ABP across loads. Results can be seen in Figure 5. Our analysis revealed that load-dependent increases in delay-period ABP were predictive of changes in the amplitude of the N1, *r*_(16)_ = −0.567, *p* = 0.042 and P3a components of the p-ER, *r*_(16)_ = −0.686, *p* = 0.001, but not the P1, *r*_(16)_ = −0.088, *p* = 0.192. Although results of the P3a analysis matched our predictions under the inhibitory view—that is, the amplitude of the P3a decreased as ABP increased—the reduction in N1 amplitude was not accompanied by an increase in delay-period ABP as expected. Instead, individuals exhibiting a larger load-dependent increase in delay-period ABP showed a smaller decrease in N1 amplitude. However, inspection of Figure 5B shows that the amplitude of the N1 was reduced in a load-dependent manner for all but a single participant, who exhibited a load-dependent increase in the N1 (see single data point in the lower-right quadrant) together with a fairly large load-dependent increase in ABP during the delay. By comparison, there appears to be no clear relationship between the load-dependent increase in ABP and the N1 for the remaining participants. Confirming this, a secondary correlation analysis revealed that the negative relationship is no longer significant when this potential outlier is removed (*r*_(1,16)_ = −0.168, *p* = 0.505). Given this, we advise caution in interpreting this particular correlation.

#### Relationship between Delay-Period ABP and Changes in K

**Figure 6:**
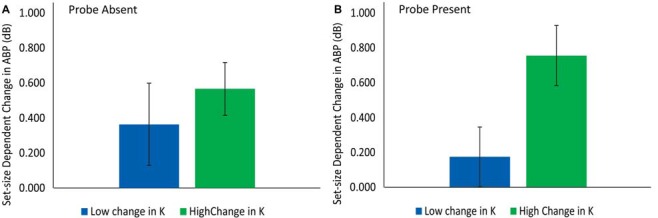
**The difference in delay-period ABP for participants with low vs. high change in K across set sizes in the probe-absent (A)** and probe-present **(B)** conditions.

Two tests were conducted to further clarify the relationship between delay-period ABP and maintenance vs. inhibitory processes. The first contrast tested the hypothesis, derived from the active maintenance view, that those individuals exhibiting the largest change in K with load, would also exhibit the largest change in delay-period ABP (1300–1400 ms). This would be the case if delay-period ABP was reflecting the storage of information in working memory *per se*. Although delay-period ABP was slightly larger for individuals exhibiting a large change in K (see Figure 6A), this difference did not approach significance, *t*_(17)_ = −0.753, *p* = 0.478. This result suggests that delay-period ABP is not directly tracking the amount of additional information that is stored in VWM across load conditions, contrary to the active processing view.

The second contrast tested the hypothesis, derived from the sensory gating view, that those individuals exhibiting a greater increase in delay-period ABP across set-sizes should be more capable of storing additional information in the probe-present condition at SS4 vs. SS2. As can be seen in Figure 6B, delay-period ABP was considerably higher for those participants exhibiting a large vs. a small increase in K from SS2 to SS4 (K_ss4_ − K_ss2_); i.e., for those who were able to store additional items in spite of the probe presentation. Confirming this, a *t*-test revealed significantly greater delay-period ABP for those individuals showing the greatest increase in the number of additional items successfully stored in the SS4 vs. SS2 conditions with a disruptive visual probe present, *t*_(17)_ = 2.37, *p =* 0.030, *d* = 0.56. This finding is consistent with the proposal that delay-period ABP serves to insulate items in working memory against probe-related disruptions.

#### ISPC

**Figure 7:**
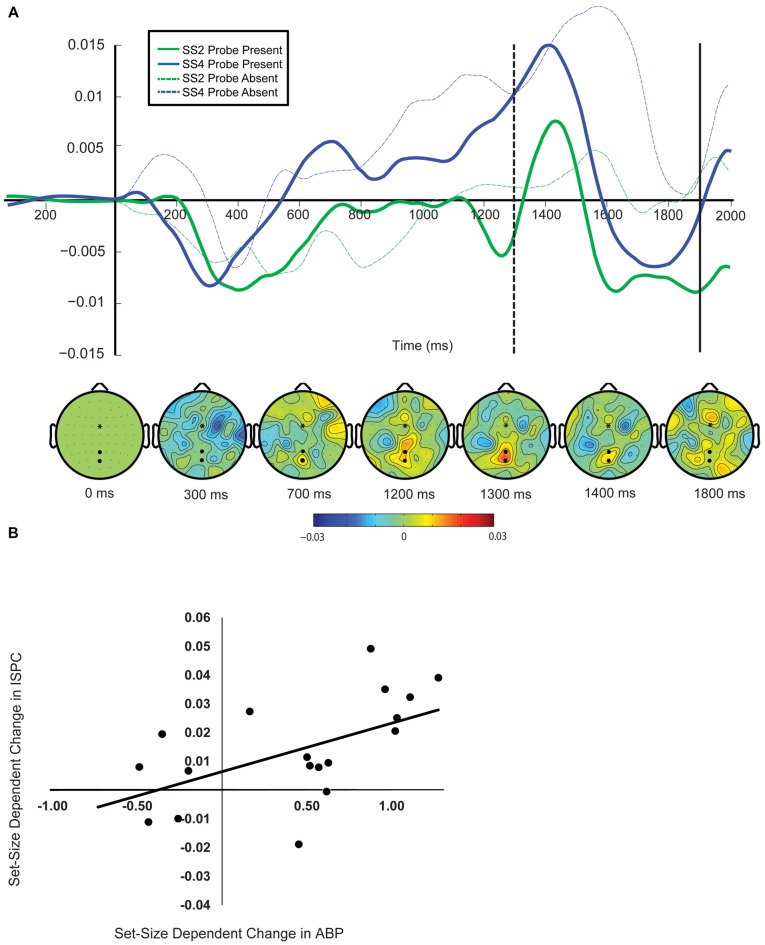
**Inter-site phase clustering (ISPC). (A)** ISPC averaged between FCz and POZ/PZ. The vertical axis reflects memory display onset. The dotted line reflects probe onset. The topographical plots below show the difference in the topographic distribution of ISPC across probe conditions (SS4-SS2) between FCz and all other electrodes during the delay interval. **(B)** The relationship between load-dependent change in ABP and change in ISPC magnitude prior to probe onset.

As can be seen in Figure 7A, ISPC in the probe-present condition exhibits a pattern very similar to the modulation of ABP observed across the delay interval (see Figure 3). In this case, however, the load-dependent difference emerges far earlier, and continues to increase throughout the same time window as the load-dependent differences in delay-period ABP emerge. Further mirroring the observed modulations in delay-period ABP, this increase appears to peak around the expected onset of the probe stimulus, falling off abruptly thereafter. Though ISPC estimates become highly unstable with lower trial numbers, it is worth noting that ISPC exhibits an analogous time course in the probe-absent condition; following the expected onset of the probe, ISPC remains stable, trailing off gradually later in the delay, similar to the pattern observed for delay-period ABP.

Looking at the same 100-ms interval prior to probe onset as was used for the delay-period ABP analyses, in the probe-present condition, a *t*-test confirmed that ISPC was significantly larger in the SS4 vs. SS2 condition, *t*_(17)_ = 2.91, *p* = 0.004, *d* = 0.69. Pearson’s *r* comparing the change in ISPC with the change in delay-period ABP across the pre-probe interval provides further evidence supporting the possibility that these increases are related. The load-dependent increase in ISPC predicted delay-period ABP increases during the same time interval, *r*_(16)_ = 0.527, *p = 0*.024 (Figure 7B).

## Discussion

Electrophysiological studies of attention and working memory suggest that modulations of oscillatory power in the alpha frequency band likely reflect the operation of an inhibitory process that serves to suppress potentially disruptive and/or task-irrelevant neural representations. In the context of the WM literature, however, it has been argued that the dependance of delay-period ABP upon both load and concurrent task demands implies that, in some cases, these oscillations may instead reflect the distributed network activity underlying the active maintenance processes associated with increasing demands on attention and WM related systems.

In the present study, we explored these alternatives by examining the role of delay-period ABP in mediating distractor processing during a VWM task. To do this, we conducted a series of analyses focused on determining whether load-dependent increases in delay-period ABP are associated with the maintenance of additional items in VWM, the suppression of ongoing visual processing, or both. The observed pattern of results was generally consistent with a variation of the inhibitory view, which holds that delay-period ABP supports VWM maintenance through the suppression of distractor processing at relatively late stages of stimulus processing.

In particular, results of the ERP analysis revealed that increases in delay-period ABP were not related to the suppression of early visual sensory processing, as the sensory gating hypothesis proposes. If oscillations in the alpha frequency range were reflecting processes suppressing early probe processing we would have expected load-dependent decreases in the P1 and N1 to be predicted by changes in delay-period ABP, as these components have been associated with initial stimulus processing and have been localized to primary and extrastriate visual areas (Di Russo et al., 2002). Contrary to this possibility, correlation analysis suggested that modulations of delay-period ABP were not predictive of changes in the P1. Additionally, although increasing load produced both an increase in delay-period ABP and a decrease in the N1, when a potential outlier was removed, these two effects were not correlated at the individual subject level. Nonetheless, the fact that these two effects were both sensitive to WM load suggests that the relationship between delay-period ABP and the N1 would be worth exploring in future research.

More in keeping with our predictions, correlation analysis showed that the observed load-dependent increase in delay-period ABP was associated with a decrease in amplitude of the P3a. Previous evidence suggests that the P3a is associated with neural activity related to the involuntary orienting of attention to novel changes in the environment (see review in Polich, 2007). Modulations of the P3a are commonly observed in the context of oddball experiments, in which relatively infrequent, task-irrelevant “deviant” stimuli are intermixed with the presentation of attended standard and target stimuli. Although the present study is quite different from a typical oddball experiment, one plausible interpretation of the observed load-dependent modulation, which is consistent with the functional interpretation of the P3a described by Polich (see also Folstein and Van Petten, 2008), is that the amplitude of the P3a tracks the extent to which putatively frontally mediated attentional resources are diverted to the task-irrelevant probe stimulus. That is, the P3a may be sensitive to the involuntary allocation of attentional resources to the probe, perhaps in an effort to distinguish it from the expected test display that appears at the end of each trial. A higher amplitude P3a would suggest greater attentive processing. If increased delay-period ABP serves to inhibit such processing, then we would expect higher delay-period ABP to be predictive of a lower amplitude P3a, which is exactly what was found. However, given the novelty of the task context, further work would be useful to confirm whether the observed ERP does indeed reflect the standard P3a measured within more common experimental paradigms.

Interestingly, other work suggests that separate sub-components of the P300 complex (P3a and P3b) may jointly reflect the reallocation of a top-down control signal responsible for selectively inhibiting extraneous brain activation when a visual stimulus becomes the focus of attention (Polich, 2007). It is possible that increased delay-period ABP observed in the current task might be reflecting the operation of this inhibitory mechanism. Lending further support to this line of reasoning, the latency of the somewhat later and more posterior P3b sub-component of the P300 has been shown to predict the simultaneous desynchronization of oscillatory activity within the alpha frequency range (8–12 Hz; Yordanova et al., 2001). Although we did not explicitly investigate this relationship, it is worth noting that we do observe a clear desynchronization in both ISPC and posterior delay-period ABP within this same time range. It is therefore possible that the P300 reflects the redistribution of the same inhibitory mechanisms underlying delay-period ABP in this task.

In addition to the ERP analyses, analyses looking at the relationship between load-dependent increases in delay-period ABP and changes in behavior provide further evidence suggesting that delay-period ABP reflects an inhibitory process that serves to protect the contents of VWM from interference, rather than reflecting maintenance *per se*. Specifically, the median-split analysis revealed that, in the probe-*absent* condition, the increase in ABP did not significantly differ between individuals showing a relatively large vs. small change in capacity across set sizes. If increased delay-period ABP was directly related to target maintenance, we would have expected it to scale as a function of the number of additional items stored in the SS4 vs. SS2 condition in the absence of the probe. Instead, ABP during the delay was found to be larger for those individuals exhibiting the greatest increase in VWM capacity from SS2 to SS4 in the probe-*present* condition. This finding supports the proposal that increased delay-period ABP tracks the participants’ ability to shield additional memory items from disruption by the probe stimulus. If such increases reflect an individual’s attempt to insulate additional maintained items against interference, then when no probe is presented, the degree to which one has implemented this strategy does little to differentially improve performance. In other words, increased distractor suppression does not help you when no distractors are presented. From this perspective, it is not surprising that delay-period ABP and increased capacity are significantly correlated in the probe-present condition only.

Finally, evidence from attention and WM tasks suggests that inhibitory control functions likely rely on interactions between frontal and parietal cortical regions (Aron et al., 2003; Samaha et al., 2015; de Pesters et al., 2016). Oscillations in the alpha frequency band have been proposed to play a critical role in this process by coordinating activity within distant cell assemblies via inter-area synchronization. This possibility was supported in the present study by the finding of a significant correlation between set-size dependent increases in delay-period ABP and phase coupling (ISPC) between frontal electrode sites and posterior electrode sites where load-dependent increases in delay-period ABP were observed. The temporal dynamics of both delay-period ABP and ISPC throughout the delay lend further support to this possibility. For example, as can be seen in Figures 3, 7, in the probe-present condition, both ISPC between frontal and posterior electrode sites and delay-period ABP at posterior electrodes increased markedly just prior to probe onset. This is what would be expected if this form of coupling and the associated increase in ABP were playing a role in mitigating the potential disruptive effects of the probe. Similarly, in the probe-absent condition (see Figures 3A, 7A), both ISPC and delay-period ABP peak around the time that the probe would be expected to appear, trailing off gradually thereafter. This suggests that inter-areal coupling and ABP may be increasing in anticipation of a potential distractor stimulus, only dissipating once it is clear that no probe will appear on that trial. This is consistent with previous evidence suggesting that the phase of oscillations in the alpha frequency band may be modulated by temporal expectations regarding stimulus onset (Jensen and Mazaheri, 2010; Samaha et al., 2015). Further, it has been shown that the amplitude of the later P300 subcomponent may be reduced selectively for stimuli that occur with a more predictable onset latency as compared to those that do not (Polich, 2007). Accordingly, we would expect any measure reflecting the mechanism responsible for suppressing the P300, in this case delay-period ABP and ISPC, to exhibit this same type of predictive temporal selectivity.

## Conclusion

In the present study, we used EEG to assess two different proposals regarding the functional role of elevated delay-period ABP in supporting the short-term retention of information in VWM. Results of both behavioral and EEG analyses supported the predictions of an inhibitory view, which holds that load-dependent increases in delay-period ABP reflect the engagement of neural processes that serve to facilitate short-term retention by inhibiting the attentive processing of task-irrelevant visual stimuli. Additionally, analysis of oscillatory phase synchronization between frontal and posterior electrode sites suggested that increased delay-period ABP may depend on coordinated activity between frontal and posterior cortical regions during maintenance. Future work in this area could benefit by the use of causal methods to examine the extent to which disrupted frontal-posterior connectivity increases interference by salient visual distractors. Additionally, it would be interesting to investigate the extent to which inhibitory activity is distributed on the basis of broad task demands, vs. as a function of the relationship between the visual properties of distracting stimuli and the contents of VWM.

## Author Contributions

AJH and JSJ developed the study concept and design; drafted the manuscript; approved the final version of the manuscript for submission. AJH collected the data and performed the data analysis and interpretation in collaboration.

## Conflict of Interest Statement

The authors declare that the research was conducted in the absence of any commercial or financial relationships that could be construed as a potential conflict of interest.
